# SARS-CoV-2 seroprevalence in healthcare workers in a high-volume ophthalmology centre in Guatemala

**DOI:** 10.1080/07853890.2021.1993325

**Published:** 2021-11-02

**Authors:** Pamela Davila-Siliezar, Alfonso Wer, Joaquin Barnoya

**Affiliations:** Department of Ophthalmology, Unidad Nacional de Oftalmología, Guatemala City, Guatemala

**Keywords:** Anti-SARS-CoV-2 antibodies, seroprevalence, COVID-19, ophthalmology, healthcare workers

## Abstract

**Purpose:**

To determine the seroprevalence of SARS-CoV-2 antibodies in eye healthcare workers (EHCW) in the largest ophthalmology centre in Guatemala and factors associated with antibody positivity.

**Methods:**

We conducted a cross sectional sero-survey in all the staff at the largest ophthalmology centre in Guatemala. Serum samples were collected and tested for total antibodies against SARS-CoV-2 employing Roche Elecsys Anti-SARS-CoV-2 Immunoassay. Results were reported as reactive or non-reactive. According to patient exposure the staff were divided into low risk (technicians, domestic and administrative staff) and high risk (nurses, ophthalmologists, anaesthesiologists, and optometrists). Among those with positive antibodies, they were given a survey that included demographic characteristics, COVID-19 exposure, and related symptomatology. Logistic regression was used to determine the factors associated with antibody positivity.

**Results:**

On November 25th a total of 94 healthcare workers were sero-surveyed, mean age was 34.15 years (±8.41), most (57.44%) were females. Seroprevalence was 18%, the majority (77%) were in the low-risk group; while 64% at high-risk, tested negative. Those at low exposure, were five times more likely to have antibodies than those at high exposure (OR:5.69; 95% CI 1.69–19.13). Age and gender were not associated to seropositivity.

**Conclusions:**

We found a similar seroprevalence of SARS-CoV-2 antibodies in EHCW to what has been reported in other healthcare groups. Seropositivity was higher among HCW with fewer patient exposure, hence the probability of community transmission.

**Key messages**
Even though eye healthcare workers are believed to be at higher risk of infection, the prevalence of antibodies against SARS-CoV-2 in this group is comparable to what has been reported previously in other healthcare groups.

## Introduction

Coronavirus disease 2019 (COVID-19) caused by severe acute respiratory syndrome coronavirus-2 (SARS-CoV-2) is a major threat to healthcare workers (HCW) since transmission is through respiratory droplets or contact with infected secretions [1].

Ophthalmologists and eye health care workers (EHCW) are believed to be at higher risk of infection due to proximity to the patients, high-volume clinics, direct contact with mucosal surfaces and secretions [2–4]. Applying preventive measures and appropriate use of personal protection equipment (PPE) is imperative to reduce the risk of infection [5,6].

Indirect detection of antibodies can be a useful tool when combined with PCR to enhance the detection of the disease [6–10]. Total antibodies are also the most sensitive and earliest serological marker, levels of which begin to increase from the second week of symptom onset [9,10]. For an effective public health response, the WHO recommends population based sero-epidemiological studies, since detection of the proportion of people with positive antibodies gives a better understanding of true extent of the disease. Population studies conducted in the United States, India, Austria, and China, had revealed a seroprevalence of 10%, 3.8%, 3.2% and 0.73% respectively [11–14].

In Guatemala the first case of COVID-19 was diagnosed in March 2020, a few days later schools and outpatient clinics closed, public and ground transportation was banned, and it was establishment a nationwide quarantine and curfew. As of February 2021, there were 158,335 confirmed cases and 5,582 deaths. There is no data of the sero-prevalence of COVID-19 in the general population in Guatemala, nor in HCW, therefore we sought to provide data on HCW in a high-volume institution, the National Ophthalmology Unit (UNO for its acronym in Spanish).

## Materials and methods

We conducted a cross-sectional sero-survey in all staff working at UNO. Blood samples were collected on 25 November 2020 and transported to Roosevelt Hospital laboratory. After centrifugation, samples were tested for total antibodies against SARS-CoV-2 employing the Elecsys® Anti-SARS-CoV-2 immunoassay (Roche, Rotkeruz, Switzerland). The immunoassay detects IgG, IgA and IgM antibodies against SARS-CoV-2 in serum and plasma (sensibility of 100% and specificity of 99.81%) using a double-antigen sandwich test principle and a recombinant protein presenting the nucleocapsid antigen. Results were reported as numeric values with a cut-off index (COI) as well as non-reactive (COI < 1.0, negative) or reactive (COI ≥ 1.0, positive). A structured questionnaire was conducted, to assess demographics, household and socioeconomic characteristics, COVID-19 associated symptoms, contacts, and previous SARS-CoV-2 PCR tests. According to patient exposure staff were divided into low and high risk. High risk defined by proximity to patients <3 ft, direct contact with patient’s mucosa and ≥2 min spent with the patient. The low risk EHCW were technicians, administrative and domestic staff, and high risk EHCW were ophthalmologists, anasthesiologists, optometrists, and nurses.

Data analysis was done using SPSS software. Continuous variables were presented as mean and standard deviation (SD) and analysed using ANNOVA. Categorical variables were summarized using percentages and analysed using chi-square test. The study was approved by the Institutional Review Board of UNO (1-2020). All patients gave written informed consent to participate in the study.

## Results

Out of 97 HCW at UNO, a total of 94 participated (97% response rate). Mean age was 34.15 years (SD 8.41) and most (57.44%) were female (Table 1). Seroprevalence for SARS-CoV-2 antibody was 18%. Among those with antibodies, 13 (77%) were low risk. More than half (53%) were administrative staff who self-reported not wearing masks while having a break from work.

**Table 1.** Socio-demographic characteristics of UNO staff N: 94.

Socio-demographic characteristics	Total of subjects	Negative antibody test n:77	Positive antibody test n:17	*p* value
Mean age, years (SD)	34.15 (8.41)	34.38 (8.09)	33.18 (9.93)	.60
Gender % (n)				
Female	57.44 (54)	57.14 (44)	58.82 (10)	–
Occupation % (n)				
*High exposure*				
Nurses	8.51 (8)	5.19 (4)	23.53 (4)	–
Doctors and optometrists	47.87 (45)	58.44 (45)	0 (0)	–
*Low exposure*				
Technicians	2.13 (2)	1.29 (1)	5.88 (1)	–
Administrative staff	34.04 (32)	29.87 (23)	52.94 (9)	–
Domestic staff	7.45 (7)	5.19 (4)	17.65 (3)	–
Risk level % (n)				
High	56.38 (53)	63.63 (49)	23.53 (4)	–
Low	43.62 (41)	36.36 (28)	76.47 (13)	–

UNO: Unidad Nacional de Oftalmologia; SD: Standard deviation.

In the same seropositive group, 12 (71%) subjects self-reported history of respiratory symptoms associated with COVID-19. Seven (41%) EHCW had a previous positive PCR or antigen test. Close contact with a confirmed COVID-19 case was reported in 10 (59%). Only 1 of the subjects was hospitalised for moderate disease.

EHCW with low exposure, were five times more likely to have antibodies than those with high exposure (OR:5.69; 95% CI: 1.69–19.13). Age and gender were not associated to seropositivity (Table 2).

**Table 2.** Socio-demographic factors associated with antibody positivity N: 94.

Socio-demographic characteristics	Positive antibody test n:17	Negative antibody test n:77	Odds ratio (95% CI)
Age (yr)			
23–45	10	71	–
48–88	1	6	1.18 (0.02–1.87)
Gender (n)			
Male	7	33	0.93 (0.14–2.71)
Female	10	44	–
High exposure % (n)			
Yes	23 (4)	64 (49)	0.17 (0.05–0.59)
No	77 (13)	36 (28)	5.69 (1.69–19.13)

CI: Confidence interval.

## Discussion

According to our findings, seropositivity of SARS-CoV-2 in EHCW is similar to that reported elsewhere [1,3,4,15]. Although ophthalmologists, anasthesiologists, optometrists and nurses are in theory at higher risk of infection because of greater exposure to patients, contact precaution and appropriate use of PPE at all time, can significantly reduce the risk of infection [2,7,15,16,17]. After the first case was diagnosed in Guatemala, the UNO adopted general measures in order to mitigate the risk of infection. These included: a 60% reduction in patient volume, reduction of staff to 50%, limiting working hours, and environmental precautions. Staff were provided with disposable gloves, surgical gowns, surgical caps, N95 masks and eye protection. In addition, polycarbonate protectors were mounted to the slit lamp and phoropters, direct ophthalmoscopy and pneumotonometry was avoided. Urgent consultations were prioritized over routine check-ups and pre-surgical COVID-19 testing was required for patients. The ophthalmology residency program shifted from traditional to virtual lectures. Live surgical training for residents was paused during the first 6 months of the pandemic.

We believe that adhering to these measures positively impacted the rate of infection among the high exposure group in our study, since we found that the risk of seropositivity in the high-risk group was much lower than that for staff at low risk. Ophthalmologists are used to deal with seemingly well clinic patients that could harbor asymptomatic COVID-19, hence their guard could be lowered, allowing the opportunity for transmission; nevertheless, they all confirmed not taking their masks off during working hours. EHCW with low exposure to patients were more prone to infection, this could be explained by community transmission. Table 3 demonstrates that low risk EHCW are more likely to public tranportation, although the difference in use between high risk EHCW and low risk EHCW was not statistically significant. Household crowding actually more common among high risk EHCW, indicating that this is not a reason for increased infeccion in low risk EHCW.

**Table 3.** Socioeconomic characteristics of EHCW divided into high and low risk of exposure.

Variables *n*	High risk EHCW	Low risk EHCW	*P* value
	(n:55)	(n:38)	
*Transportation*			
Private	42 (76%)	26 (68%)	.39
Public	13 (24%)	12 (32%)	–
*Overcrowded household* ^a^			
No	0 (0%)	6 (16%)	.01
Yes	55 (100%)	32 (84%)	–
*Higher education level* ^b^			
yes	52 (95%)	26 (68%)	.0008
no	3 (5%)	12 (32%)	–

EHCW: eye healthcare workers.

^a^Three or more in the same bedroom, ^b^Greater than high school.

High participation of the staff is a strength of our study, for it can give a general idea of COVID-19 situation among HCW in low-middle income countries with shortage of PCR tests. We can conclude that the risk of infection amongst healthcare workers appear to be partly related to working with sick patients, but to a greater extent to their social behavior.

## References

[1] Remuzzi A, Remuzzi G. COVID-19: protecting health-care workers. Lancet. 2020;395(10231):1225–1228. 32178769 10.1016/S0140-6736(20)30627-9 PMC7102589

[2] Breazzano MP, Shen J, Abdelhakim AH,et al. New York city COVID-19 resident physician exposure during exponential phase of pandemic. J Clin Invest. 2020;130(9):4726–4733. 32463802 10.1172/JCI139587 PMC7456242

[3] Chang D, Xu H, Rebaza A, et al. Protecting health-care workers from subclinical coronavirus infection. Lancet Respir Med. 2020;8(3):e13. 32061333 10.1016/S2213-2600(20)30066-7 PMC7128440

[4] Danesh-Meyer H, McGhee C. Implications of COVID-19 for ophthalmologists. Am J Ophthalmol. 2020;223:108–118. 32976847 10.1016/j.ajo.2020.09.027 PMC7506460

[5] Wan K, Huang S, Young A, et al. Precautionary measures needed for ophthalmologists during pandemic of the coronavirus disease 2019 (COVID-19). Acta Ophthalmol. 2020;98(3):221–222. 32223068 10.1111/aos.14438 PMC7540674

[6] Li O, Shantha J, Wong T, et al. Preparedness among ophthalmologists: during and beyond the COVID-19 pandemic. Ophthalmology. 2020; 127(5):569–572. 32327128 10.1016/j.ophtha.2020.03.037 PMC7167498

[7] Guo L, Ren L, Yang S, et al. Profiling early humoral response to diagnose novel coronavirus disease (COVID-19). Clin Infect Dis. 2020;71(15):778–785. 32198501 10.1093/cid/ciaa310 PMC7184472

[8] Li G, Fan Y, Lai Y, et al. Coronavirus infections and immune responses. J Med Virol. 2020;92(4):424–432. 31981224 10.1002/jmv.25685 PMC7166547

[9] Sethuraman N, Stanleyraj S, Ryo A. Interpreting diagnostic tests for SARS-CoV-2. JAMA. 2020;323(22):2249–2251. 32374370 10.1001/jama.2020.8259

[10] Zhao J, Yuan Q, Wang H, et al. Antibody responses to SARS-CoV-2 in patients of novel coronavirus disease 2019. Clin Infect Dis. 2020;71(16):2027–2034. 32221519 10.1093/cid/ciaa344 PMC7184337

[11] Murhekar M, Bhatnagar T, Selvaraju S, et al. Prevalence of SARS-CoV-2 infection in India: findings from the national serosurvey, May-June 2020. Indian J Med Res. 2020;152(12):48–60. 32952144 10.4103/ijmr.IJMR_3290_20 PMC7853249

[12] Fuereder T, Berghoff A, Heller G, et al. SARS-CoV-2 seroprevalence in oncology healthcare professionals and patients with cancer at a tertiary care center during the COVID-19 pandemic. ESMO Open. 2020;5(5):e000889. 32878898 10.1136/esmoopen-2020-000889 PMC7470513

[13] Bajema K, Wiegand R, Patel S, et al. Estimated SARS-CoV-2 seroprevalence in the US as of September 2020. JAMA Intern Med. 2021;181(4):450. 33231628 10.1001/jamainternmed.2020.7976 PMC7686880

[14] Xu X, Sun J, Nie S, et al. Seroprevalence of immunoglobulin M and G antibodies against SARS-CoV-2 in China. Nat Med. 2020;26(8):1193–1195. 32504052 10.1038/s41591-020-0949-6

[15] Seto W, Tsang D, Yung R, et al. Effectiveness of precautions against droplets and contact in prevention of nosocomial transmission of severe acute respiratory syndrome (SARS). Lancet. 2003;361(9368):1519–1520. 12737864 10.1016/S0140-6736(03)13168-6 PMC7112437

[16] Chee S, Lun K. SARS: a timely reminder. Br J Ophthalmol. 2013;97(9):1217–1218. 23793905 10.1136/bjophthalmol-2013-303596

[17] Yu I, Oon K, Songbo P. Perspectives on coronavirus disease 2019 control measures for ophthalmology clinics based on a Singapore center experience. JAMA Ophthalmol. 2020;138(5):435–436. 32232466 10.1001/jamaophthalmol.2020.1288

